# SARS-CoV-2 B.1.1.7 Decline Is Not Driven by the Introduction of a More Successful Variant

**DOI:** 10.1128/Spectrum.01128-21

**Published:** 2021-11-17

**Authors:** Cristina Rodríguez-Grande, Sergio Buenestado-Serrano, Luis Alcalá, Pedro J. Sola-Campoy, Andrea Molero-Salinas, Álvaro Otero-Sobrino, Jorge Rodríguez-Grande, Víctor Manuel de la Cueva García, Javier Adán-Jiménez, Carla Rico-Luna, Carmen Losada, Pilar Catalán, Patricia Muñoz, Laura Pérez-Lago, Darío García de Viedma

**Affiliations:** a Servicio de Microbiología Clínica y Enfermedades Infecciosas, Gregorio Marañón General University Hospital, Madrid, Spain; b Instituto de Investigación Sanitaria Gregorio Marañón (IiSGM), Madrid, Spain; c CIBER Enfermedades Respiratorias (CIBERES), Madrid, Spain; d Departamento de Medicina, Universidad Complutense, Madrid, Spain; Peking University People’s Hospital

**Keywords:** COVID-19, variants of concern, Spain, SARS-CoV-2

## Abstract

The SARS-CoV-2 variant of concern (VOC) Delta (B.617.2 lineage) displaced the predominant VOC Alpha (B.1.1.7 lineage) in the United Kingdom. In Madrid, recent start of the decline of predominant VOC Alpha suggested an equivalent phenomenon. However, 11 different variants, none overrepresented in frequency, occupied progressively over a period of 7 weeks the niche previously dominated by VOC Alpha. Only after these 7 weeks, VOC Delta started to emerge. Viral competition due to the entry of VOC Delta is not the major force driving the start of VOC Alpha decline in Madrid.

**IMPORTANCE** Our data indicate that the dynamics of SARS-CoV-2 VOCs turnover in our setting differ from those proposed for other countries. A systematic genomic analysis, updated on a weekly basis, of representative randomly selected samples of SARS-CoV-2 circulating variants allowed us to define a lapse of 7 weeks between the start of VOC Alpha decline and the final emergence of VOC Delta. During this period, VOC Alpha showed a sustained decline, while 11 VOCs, variants of interest (VOIs), and other identified variants, none overrepresented, occupied the niche left by VOC Alpha. Only after these 7 weeks, emergence of VOC Delta occurred, indicating that viral competition involving VOC Delta was not the exclusive direct driving force behind the starting of VOC Alpha decline.

## OBSERVATION

Several SARS-CoV-2 variants of concern (VOCs) have emerged along the COVID-19 pandemic. VOCs were initially described in the United Kingdom (B.1.1.7, VOC Alpha), South Africa (B.1.351, VOC Beta), Brazil (P.1, VOC Gamma), and recently, in India (B.1.617.2, VOC Delta) (1) (https://cov-lineages.org/lineages/lineage_B.1.617.html). These variants accumulate mutations with potential significance, particularly when occurring in the gene coding for the spike protein or even in the receptor-binding domain. The constellation of relevant mutations raised the alarm on their role in enhancing SARS-CoV-2 transmissibility, reducing immune neutralization or vaccine-mediated protection (2). Genomic-based surveillance has been instrumental to track VOCs worldwide, identify their introduction to different countries, and monitor subsequent spreading.

The first described VOC, namely, VOC Alpha, has greater transmission capacity, which caused 97.2% (February 2021) of new SARS-CoV-2 infections in England to be associated with this variant in few months, later spreading to at least 141 countries (https://www.gov.uk/government/publications/investigation-of-novel-sars-cov-2-variant-variant-of-concern-20201201; https://cov-lineages.org/global_report_B.1.1.7.html). This epidemiological scenario in the United Kingdom, dominated by VOC Alpha, has recently changed due to another VOC, Delta, responsible for a complete turnover of the leading variant and currently associated with 99.7% of all the cases in the United Kingdom (https://www.gov.uk/government/publications/investigation-of-novel-sars-cov-2-variant-variant-of-concern-20201201).

**FIG 1:**
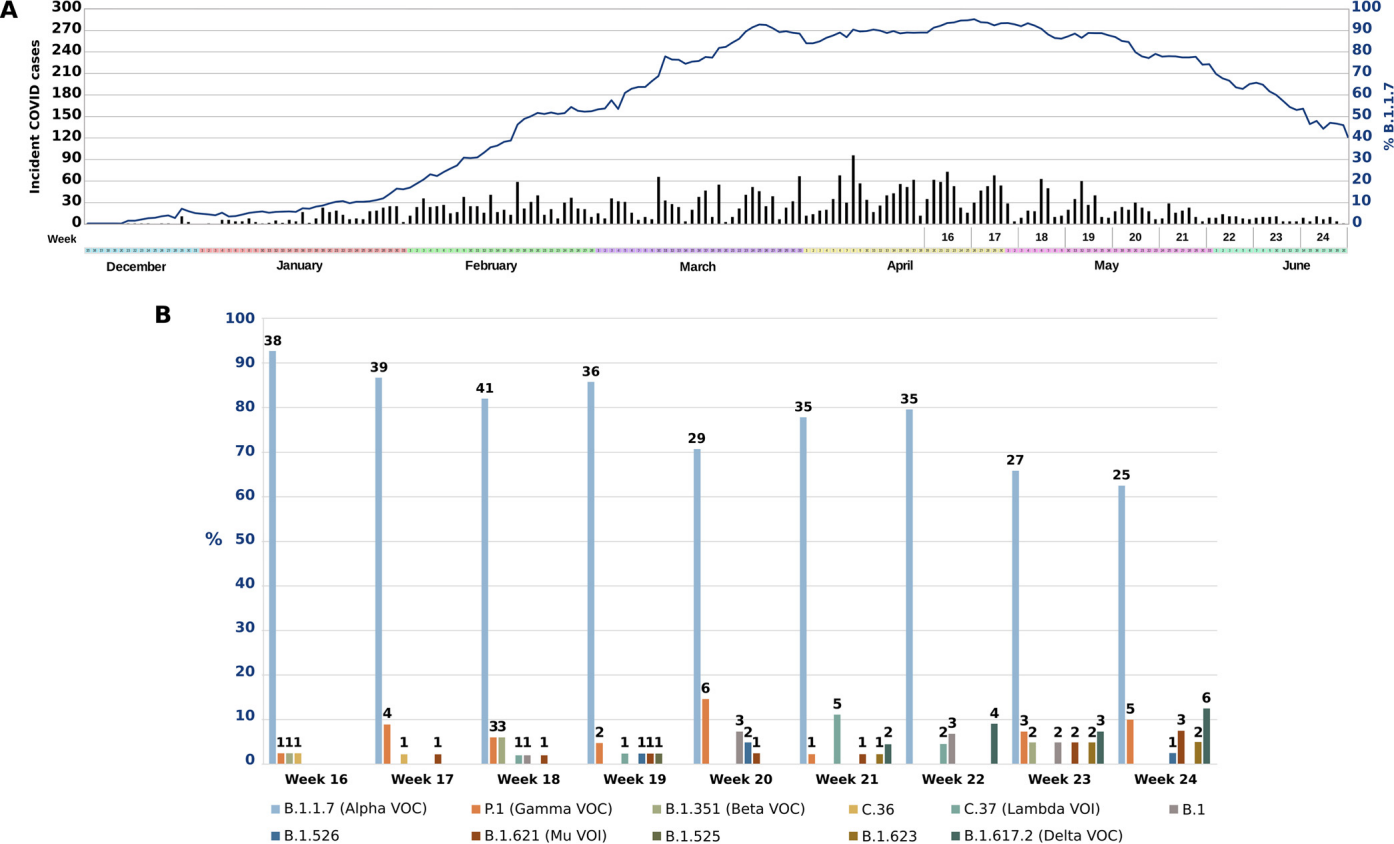
(A) Weekly VOC Alpha frequency based on TaqPath data from all newly diagnosed COVID-19 cases. (B) Weekly VOC Alpha and other SARS-CoV-2 variants frequency based on whole-genome sequencing of the randomly selected sample. The numbers on top of bars correspond to the number of cases for each variant.

For the diagnosis of COVID-19 by reverse transcriptase PCR (RT-PCR) among our population in Madrid, we used the TaqPath test (Thermo Fisher Scientific, USA). This test allows for the determination of population-based frequency of VOC Alpha, taking advantage of the S gene dropout (https://www.ecdc.europa.eu/en/publications-data/threat-assessment-brief-rapid-increase-sars-cov-2-variant-united-kingdom), a way to indicate this VOC. TaqPath results confirmed the same situation reported in the United Kingdom, with all cases associated with this VOC by 18 March, 13 weeks after the description of the first cases in mid-December 2020 (3) (Fig. 1A). VOC Alpha remained dominant (>80% over total population, calculated as weekly moving average) from week 11 to week 20, starting to decline around 25 April (week 16) (global maximum in Alpha VOC relative frequency could be found by adjusting the time series to a susceptible-infected-recovered (SIR) model, as stated in reference 4, and a locally weighted scatterplot smoothing (LOWESS) regression curve, whose parameters always rendered global maximum around days 24 to 27 April—weeks 16 to 17—regardless of how conservative the fit was). These dynamics led us to evaluate if VOC Alpha was being displaced by a more successfully transmitted VOC, VOC Delta, as has been described in the United Kingdom (https://www.gov.uk/government/publications/investigation-of-novel-sars-cov-2-variant-variant-of-concern-20201201).

Surveillance of SARS-CoV-2 variants circulating among our population in Madrid lies on whole-genome sequencing (WGS) analysis of a random selection of cases from all incident cases diagnosed by RT-PCR the previous week with a threshold cycle (*Ct*) of <32. Random sampling was performed using the simple randomization method (5), using computer-generated random numbers by means of Excel 2013 program. Data on the presence and distribution of VOC were updated quickly (72 h after finishing the weekly sampling). WGS was performed with the Artic_nCov-2019_V3 panel of primers. Libraries were prepared using the Nextera Flex DNA library preparation kit (Illumina Inc, CA, USA) and sequenced on the MiSeq system (Illumina Inc, CA, USA). An in-house pipeline was applied to analyze the sequencing reads (https://github.com/pedroscampoy/covid_multianalysis) (6). Data supporting the findings of this study (FASTA files) are openly available in GISAID (https://www.gisaid.org/; see Table S1 in the supplemental material).

**FIG 2:**
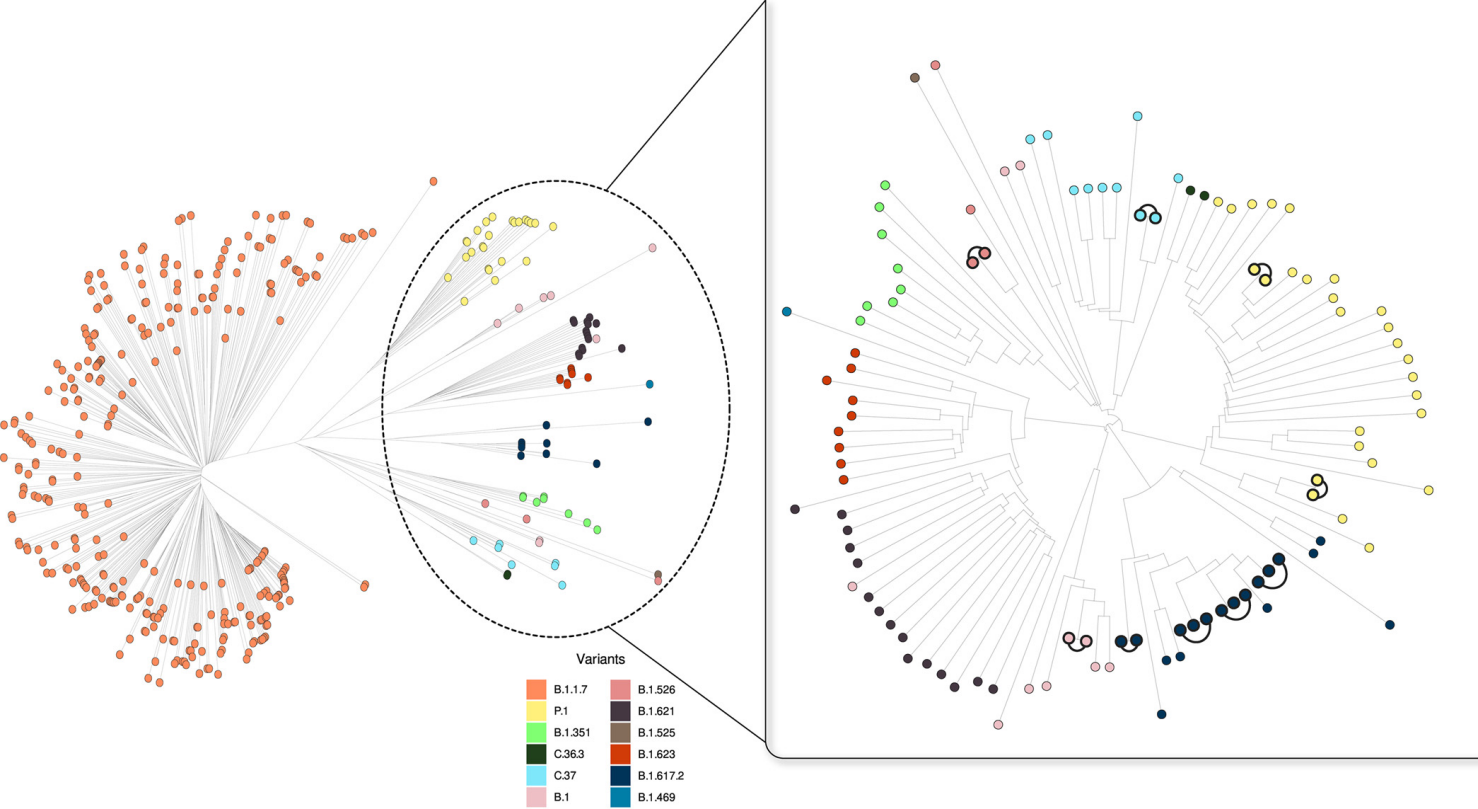
Phylogenetic tree obtained from the sequences from specimens analyzed in the study period. Sequences corresponding to non-VOC Alpha variants are zoomed in. The links in the zoomed section indicate the cases included in the same cluster.

From week 16, when the first decrease in VOC Alpha frequency was detected (TaqPath data), 45 to 50 cases (weeks 16 to 24; 14.5 to 52.3% of total diagnosed cases over these weeks) were sequenced. We must acknowledge our limited sample size, but it still allowed us to find some relevant data. First, WGS data confirmed a significant decline in VOC Alpha frequency starting at week 17 (Mann-Kendall test, weeks 16 to 24 partial frequency of Alpha VOC: tau value of −0.8; 1-sided *P* value of 0.01; Fig. 1B). The frequency dropped from 92.6% at week 16 to 60.5% at week 24. Second, we observed that the decline in these weeks was not associated with a corresponding increase of another leading variant, as described in the United Kingdom. Differently, in our setting, the population niche left available by the VOC Alpha when declining was occupied by a diversity of variants (Fig. 1B and Fig. 2). Eleven different variants other than VOC Alpha were detected during the first 7 weeks of VOC Alpha steady decline, none of them superior in frequency among the others (Fig. 1B). Distribution of these 11 variants was as follows: 52% other VOCs (Beta, Gamma, and Delta), 31% variants of interest (VOIs) (C.37, C36.3, B.1.621, B.1.525, and B.1.526 lineages), and 17% non-VOCs/non-VOIs (B.1, B.1.469, and B.1.623 lineages) (Fig. 1B). According to our data set, VOC Gamma frequency did not show the rising trend (Mann-Kendall test tau value of 0.05; 2-sided *P* value of 0.9) observed in neighboring countries, as in some regions of France, where VOC Beta and Gamma showed more successful transmissions compared to VOC Alpha (7).

VOC Delta was first detected in our analysis of random sampling by week 21 of 2021, 5 weeks after decline of VOC Alpha was observed. Over the following 3 weeks, it reached the second position in frequency (until 12.5%) with a significant growing tendency (Mann-Kendall residuals tau value of 0.8; 1-sided *P* value of 0.016).

Despite the fact that we are witnessing the initial moments of VOC Delta in our population, a higher number of clustered cases (0 single nucleotide polymorphisms [SNPs] between the cases; Fig. 2) was identified associated with VOC Delta (four clusters involving 3, 3, 3, and 2 cases each), which is consistent with higher transmissibility. The remaining clusters (Fig. 2) involved the B.1, B.1.526, and C.37 lineages (one cluster involving two cases each).

It should be noted that, at the time of submitting the manuscript, VOC Delta has increased its presence, turning into the major variant in our population in Madrid (96.2% of the cases, as per TaqPath assay, week 35 of 2021), finally reproducing the situation in the United Kingdom.

If virological competition seems not to be the direct driver for VOC Alpha decline in our setting, the alternative likely explanation is that its decline might be likely due to a differential impact of vaccination on the different SARS-CoV-2 variants. This may be considered, as response to vaccines has not been found to be diminished for VOC Alpha (8), whereas reduced protection has been found for other VOCs (8, 9). Regarding Delta VOC, it contains diverse mutations in the N-terminal domain and the receptor-binding domain of the SARS-CoV-2 spike protein, which are associated with a reduced sensitivity to antibody neutralization, as demonstrated recently (10).

### Conclusion.

Our data indicate that the dynamics of SARS-CoV-2 VOC turnover in our setting differ from those described in other European countries. A systematic genomic analysis, updated on a weekly basis, of representative randomly selected samples of SARS-CoV-2 circulating variants allowed us to define a lapse of 7 weeks between the start of VOC Alpha decline and emergence of VOC Delta. During this period, VOC Alpha showed a sustained decline, while 11 VOCs, VOIs, and other identified variants, none overrepresented, occupied the niche left by VOC Alpha. Only after these 7 weeks, clear emergence of VOC Delta occurred, indicating that viral competition involving VOC Delta was not the direct driving force behind the start of VOC Alpha decline.
